# Tandem Mass Tag-Based Quantitative Proteomics and Virulence Phenotype of Hemolymph-Treated Bacillus thuringiensis kurstaki Cells Reveal New Insights on Bacterial Pathogenesis in Insects

**DOI:** 10.1128/Spectrum.00604-21

**Published:** 2021-10-27

**Authors:** Yanyan Sun, Linlin Yang, Lianet Rodríguez-Cabrera, Yushan Ding, Chaoliang Leng, Huili Qiao, Siliang Huang, Yunchao Kan, Lunguang Yao, Denis J. Wright, Dandan Li, Camilo Ayra-Pardo

**Affiliations:** a China-UK-NYNU-RRES Joint Laboratory of Insect Biology, Henan Key Laboratory of Insect Biology in Funiu Mountain, School of Life Sciences and Agricultural Engineering, Nanyang Normal University (NYNU), Nanyang, People’s Republic of China; b Plant Division, Centre for Genetic Engineering and Biotechnology (CIGB), Havana, Cuba; c Department of Life Sciences, Faculty of Natural Sciences, Imperial College London, Ascot, United Kingdom; Howard University

**Keywords:** TMT-based quantitative proteomics, insect pathogen, septicemia, oxidative stress, virulence, biofilm formation, immune evasion, persistence, biofilms, proteomics

## Abstract

The spore-forming bacterium Bacillus thuringiensis (Bt) of the Bacillus cereus group uses toxin-opened breaches at the insect midgut epithelium to infest the hemolymph, where it can rapidly propagate despite antimicrobial host defenses and induce host death by acute septicemia. The response of Bt to host hemolymph and the latter’s role in bacterial pathogenesis is an area that needs clarification. Here, we report a proteomic analysis of the Bt *kurstaki* strain HD73 (Btk) hemolymph stimulon showing significant changes in 60 (34 up- and 26 downregulated) differentially accumulated proteins (DAPs). Gene ontology (GO) enrichment analysis revealed that DAPs were mainly related to glutamate metabolism, transketolase activity, and ATP-dependent transmembrane transport. KEGG analysis disclosed that DAPs were highly enriched in the biosynthesis of bacterial secondary metabolites, ansamycins. Interestingly, about 30% of all DAPs were *in silico* predicted as putative virulence factors. Further characterization of hemolymph effects on Btk showed enhanced autoaggregation in liquid cultures and biofilm formation in microtiter polystyrene plates. Hemolymph-exposed Btk cells were less immunogenic in mice, suggesting epitope masking of selected surface proteins. Bioassays with intrahemocoelically infected Bombyx mori larvae showed that hemolymph preexposure significantly increased Btk toxicity and reproduction within the insect (spore count per cadaver) at low inoculum doses, possibly due to ‘virulence priming’. Collectively, our findings suggest that the Btk hemolymph stimulon could be partially responsible for bacterial survival and propagation within the hemolymph of infected insects, contributing to its remarkable success as an entomopathogen. All mass spectrometry data are available via ProteomeXchange with identifier PXD021830.

**IMPORTANCE** After ingestion by a susceptible insect and damaging its midgut epithelium, the bacterium Bacillus thuringiensis (Bt) reaches the insect blood (hemolymph), where it propagates despite the host’s antimicrobial defenses and induces insect death by acute septicemia. Although the hemolymph stage of the Bt toxic pathway is determinant for the infested insects’ fate, the response of Bt to hemolymph and the latter’s role in bacterial pathogenesis has been poorly explored. In this study, we identified the bacterial proteins differentially expressed by Bt after hemolymph exposure. We found that about 30% of hemolymph-regulated Bt proteins were potential virulence factors, including manganese superoxide dismutase, a described inhibitor of hemocyte respiratory burst. Additionally, contact with hemolymph enhanced Bt virulence phenotypes, such as cell aggregation and biofilm formation, altered bacterial immunogenicity, and increased Bt toxicity to intrahemocoelically injected insects.

## INTRODUCTION

The spore-forming bacterium Bacillus thuringiensis (Bt) of the Bacillus cereus group is a well-known insect pathogen widely exploited as a microbial pesticide (1, 2). Bt strains variously produce highly specific pore-forming proteins (Cry, Cyt, Vip, and Sip) and other virulence factors positively regulated by the pleiotropic quorum sensor phospholipase C regulator (PlcR), such as phospholipases C, hemolysins, enterotoxins, and proteases to breach the insect host midgut epithelium barrier in order to infest the hemolymph (3–8). Within the hemolymph, Bt vegetative stages propagate despite antimicrobial host defenses to induce insect death by acute septicemia (9, 10). Bt can survive in the insect cadaver (necrotrophism) aided by the PlcR-regulated quorum sensor NprR, which controls the expression of metalloproteases, lipases, chitinases, and enzymes for biosynthesis and secretion of a lipopeptide kurstakin involved in biofilm formation (11, 12). Necrotrophic Bt cells ultimately sporulate under the control of transcription factor Spo0A in its phosphorylated form (Spo0A∼P) (13). Throughout the infectious process, quorum quenching enzymes and antibiosis may help Bt to outperform the other gut bacteria (14–16).

Functional expression of *plcR* is required for the activation of the PlcR virulence regulon and Bt’s pathogenicity in insects following oral infection (4, 5, 16, 17). However, the toxicity of a Δ*plcR* mutant strain was not affected in the same magnitude when intrahemocoelic injection was used as the inoculation route (4, 18), which suggests that other factors may be involved in survival and propagation of Bt within the hemolymph. Indeed, different Bt subpopulations of differentiated cells have been described to coexist throughout the infectious process, some of which are regulated by as-of-yet undefined master regulators (13, 19). The response of Bt to hemolymph and the latter’s role in bacterial pathogenesis is, therefore, an area that needs clarification.

Components of insect cellular and humoral innate responses circulate continuously in the hemolymph to prevent disease and infection (20–24). Hemocytes, such as granulocytes and plasmatocytes, mediate the cellular responses to microbial insults through phagocytosis, encapsulation, and superoxide production (25, 26). Humoral responses include melanization, clotting, and secretion of antimicrobial peptides (AMPs) and complement-like proteins to immobilize and kill the intruders (27). The activation of both responses relies on the recognition of pathogen-associated molecular patterns (PAMPs) on the microbial surface by pattern recognition receptors (26, 28). Bt can suppress insect humoral immune defenses (29); however, the process remains poorly characterized.

The best known Bt anti-immune strategy is the expression of metalloprotease InhA1, the major component of the exosporium, which is involved in *Bacillus* spore survival in macrophages and their escape from phagocytes (30–32). InhA1 has also been found digesting cecropin and attacin AMPs in the insect hemolymph (33). The expression of InhA1 is dependent on Spo0A∼P, which in turn represses PlcR (34, 35). Another Bt metalloprotease not regulated by PlcR, the cell surface-associated camelysin (CalY), was recently shown to play critical roles in both bacterial adhesions to insect cells (adhesin activity) and in biofilm formation and is required for Bt’s full virulence to insects by either ingestion or intrahemocoelic injection routes (36–38).

In the present study, we tested the hypothesis that hemolymph exposure by Bt would activate a specific response in the bacterium consisting of a change in the synthesis rate of a set of proteins that contribute to Bt’s survival and proliferation within the insect body. We investigated the effects of Bombyx mori larval hemolymph exposure on the proteome of Bt *kurstaki* strain HD73 (Btk) bacterium through tandem mass tag (TMT)-based quantitative proteomics. Additionally, we compared hemolymph-exposed and control bacteria regarding autoaggregation, biofilm formation, surface protein immunogenicity, and pathogenicity in insects. We identified a Btk hemolymph stimulon that includes an enhanced ability to form unattached bacterial aggregates in liquid cultures and attached biofilms on microtiter polystyrene plates, altered bacterial immunogenicity, and increased toxicity to intrahemocoelically injected *Bo. mori* larvae, possibly due to virulence priming.

## RESULTS

### Defining a Btk hemolymph stimulon using proteomics.

**FIG 1:**
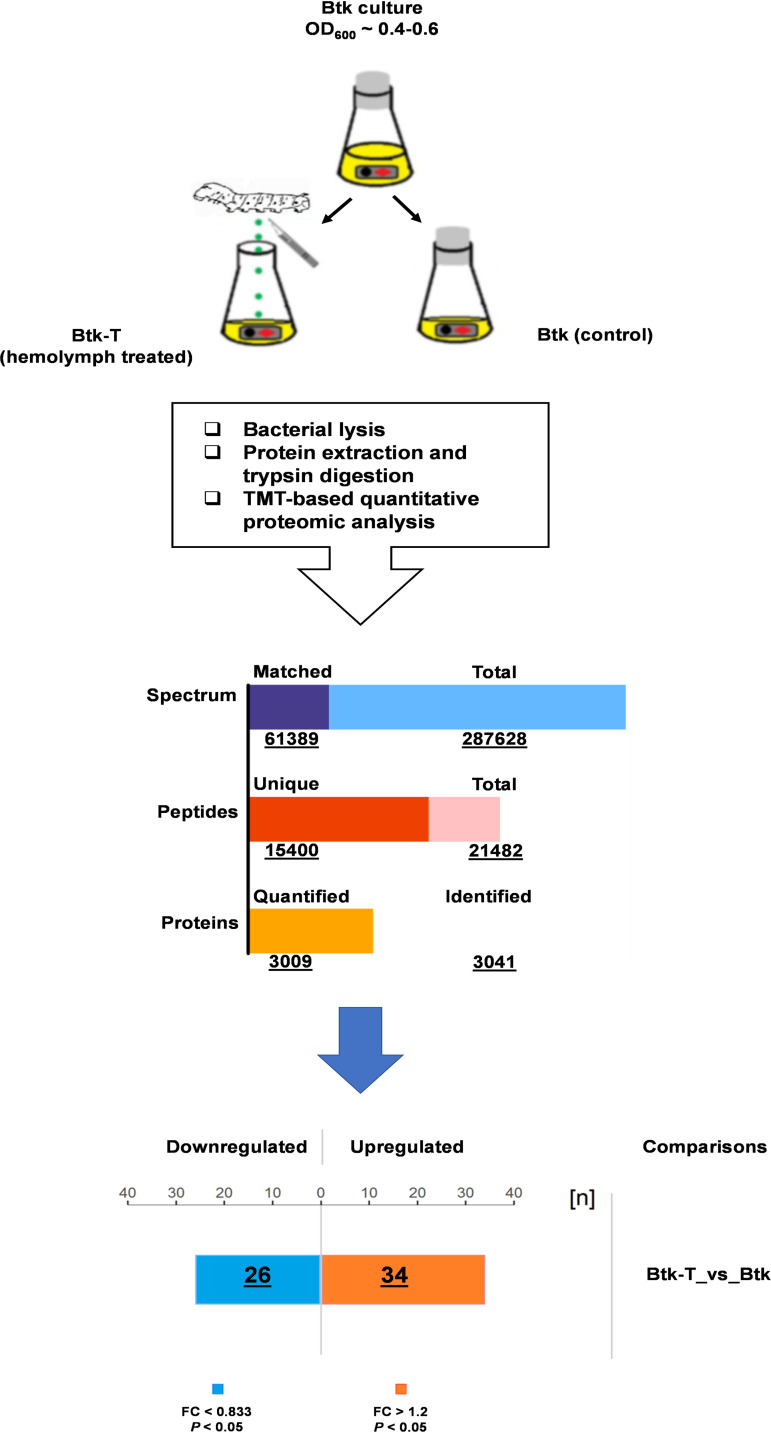
Schematic representation of the workflow followed in this study for the proteomic analysis of the Btk hemolymph stimulon. Features of TMT-based quantitative proteomic results are shown underlined; FC, fold change.

Comparative TMT-based proteomic analysis was performed to investigate changes in protein levels and affected pathways in Btk vegetative cells treated with 1:100 (vol/vol) *Bo. mori* hemolymph for 1 h (Btk-T) (Fig. 1). TMT data validation showed mass measurement error within 10 ppm, ∼81.15% of modified peptides with a MASCOT score of 20 or more (the median was 33.99), and lengths of peptides varied from 7 to 20 amino acid residues, all indicative of high-quality mass spectrophotometry (MS) experimental data (Fig. S1 and Table S1 in the supplemental material).

**FIG 2:**
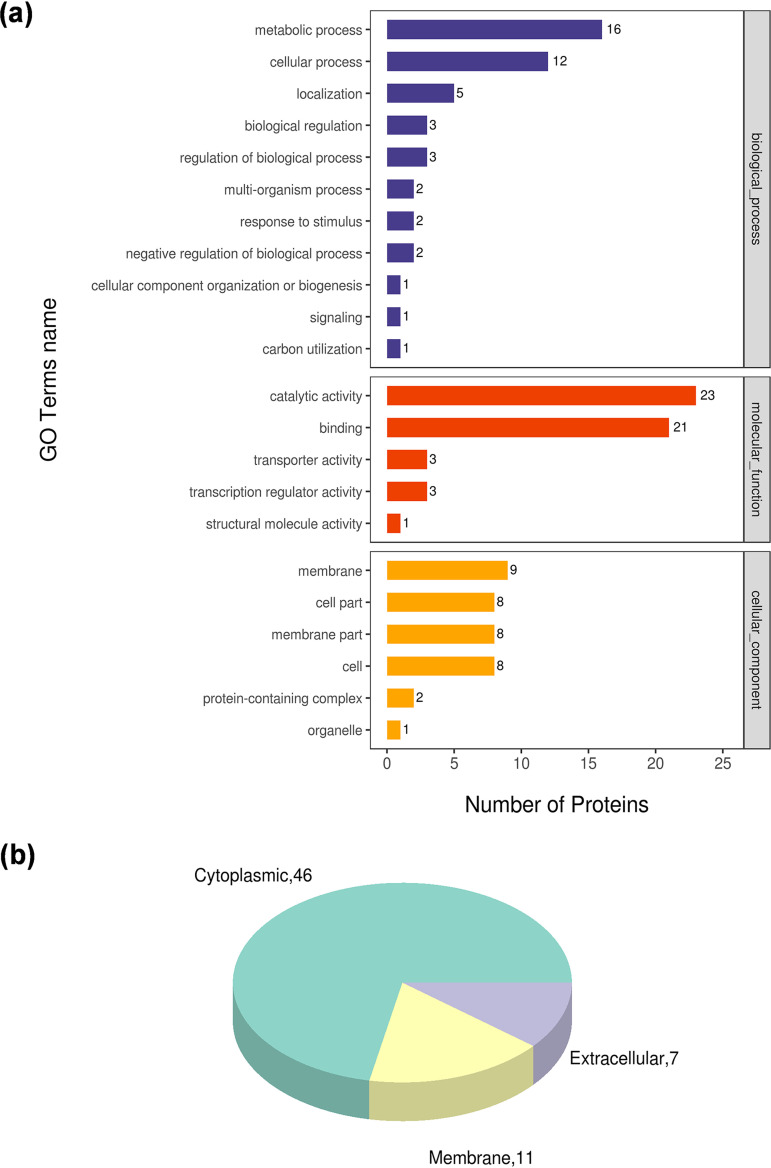
Gene ontology (GO) analysis of differentially accumulated proteins (DAPs) in the Btk hemolymph stimulon. Most of the DAPs could be classified into three categories using assigned GO terms. (a) GO level 2 functional annotation information, including “biological process,” “molecular function,” and “cellular component” categories, which are distinguished by blue, red, and orange in sequence. The abscissa is the number of DAPs under each category. (b) Subcellular localization of DAPs according to CELLO (http://cello.life.nctu.edu.tw/).

A total of 3,041 proteins were identified and annotated by using the Bt protein sequence information in the UniProt database (Table S2), 3,009 of which were quantified. Sixty proteins were significant (*P *< 0.05) differentially accumulated proteins (DAPs) with abundances that changed greater than 1.2-fold (Btk-T/Btk), of which 34 were upregulated and 26 downregulated (Table S3). Most of the DAPs could be classified into the following three categories using assigned Gene ontology (GO) terms: biological process, molecular function, and cellular component. For the biological process category, the most representative term was “metabolic process” (16 DAPs). For molecular function, “catalytic activity” was the dominant term (23 DAPs), and for cellular component, the largest term was “membrane” (9 DAPs) (Fig. 2a; Table S2). According to subcellular localization prediction, the 60 DAPs were distributed among cytoplasmic, membrane, and extracellular compartments, with some of them predicted in more than one compartment (Fig. 2b; Table S2).

**FIG 3:**
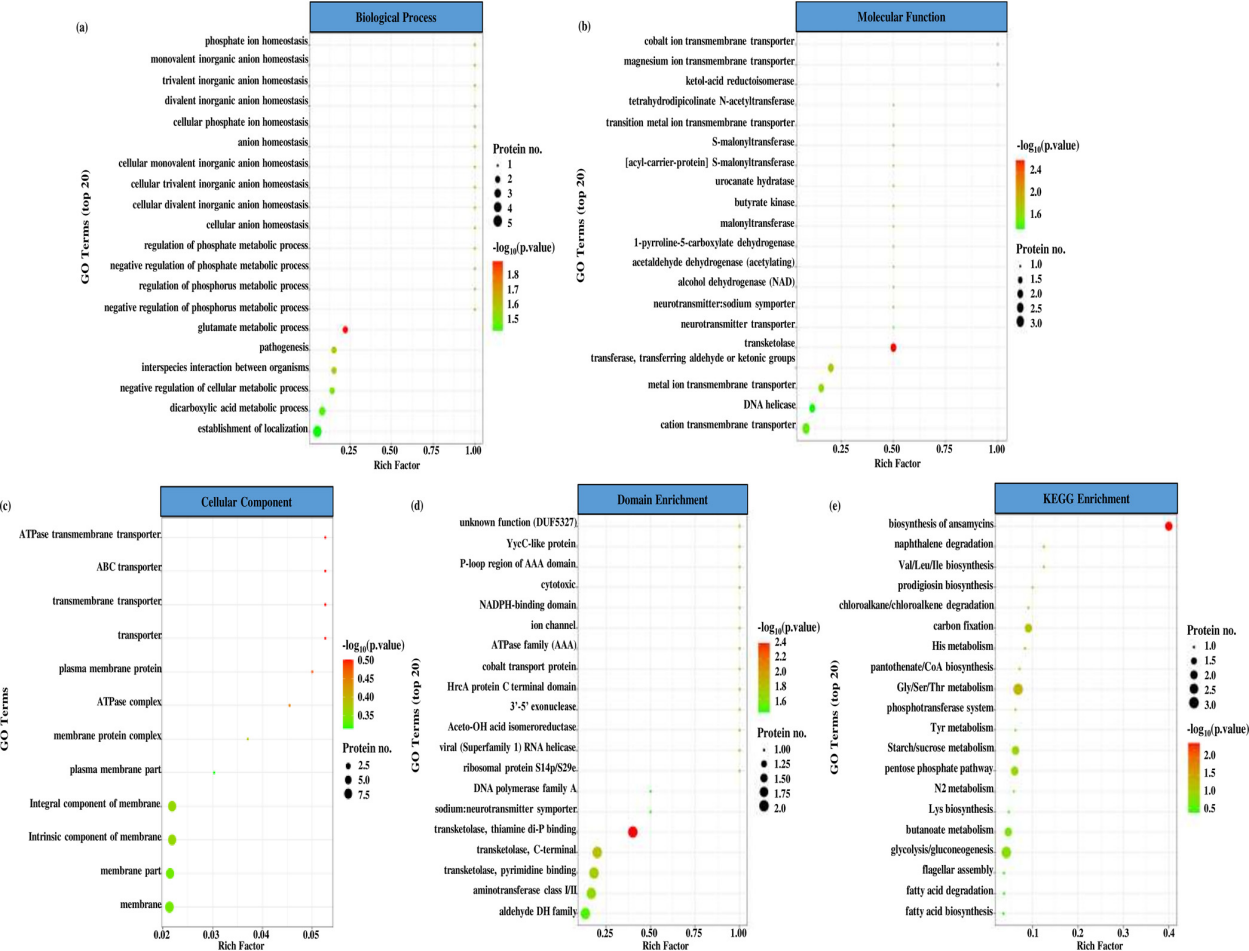
GO, protein domain, and KEGG enrichment analysis of DAPs in the Btk hemolymph stimulon. The most significantly enriched categories (*P < *0.05) revealed by the Fisher’s exact test are shown in the bubble charts. (a) Biological process. (b) Molecular function. (c) Cellular component. (d) Protein domain enrichment. (e) KEGG pathway enrichments. The ordinate of the bubble charts is the functional classification or pathway, and the abscissa is the enrichment factor (rich factor of ≤1) calculated as the ratio of the number of DAPs to the number of total annotated proteins in a certain functional class or pathway. The bubble color indicates the *P* value, and the color gradient represents the size of the *P* value, converting from −log_10_. The closer the color is to red, the lower the *P* value and the more significant the enrichment. The bubble size indicates DAP number in the functional class or pathway (the bigger bubbles refer to a larger amount).

We further analyzed the functional enrichment of the 60 DAPs at three levels, including GO classification (Table S4), protein domain (Table S5), and KEGG pathway (Table S6). Fig. 3 shows bubble charts of the most significantly enriched categories according to their *P* values that were revealed by Fisher’s exact tests. Regarding the GO classification, we found DAPs highly enriched in glutamate metabolic processes (GO:0006536) from the biological process category results (Fig. 3a), transketolase activity (GO:0004802) from the molecular function category results (Fig. 3b), and ATPase-dependent transmembrane transport complex (GO:0098533), ATP-binding cassette (ABC) transporter complex (GO:0043190), transmembrane transporter complex (GO:1902495), transporter complex (GO:1990351), plasma membrane protein complex (GO:0098797), and ATPase complex (GO:1904949) from the cellular component category results (Fig. 3c). Protein domain enrichment analysis showed DAPs enriched in the three different domains of the transketolase (TktA) enzyme (EC: 2.2.1.1), that is, “thiamine diphosphate-binding domain” (PF00456), “C-terminal domain” (PF02780), and “pyrimidine-binding domain” (PF02779), with enrichment scores decreasing in the above order (Fig. 3d). KEGG is a collection of databases for understanding biological functions, such as metabolic pathways, biomolecular complexes, and biochemical reactions (39). We found a KEGG pathway related to the biosynthesis of ansamycins (ko01051) to exhibit the highest enrichment score in DAPs (Fig. 3e).

Virulence potential of DAPs was assessed by *in silico* prediction of virulence factors using the online tool VFDB (virulence factor database; http://www.mgc.ac.cn/VFs/). We found 10 of the 34 upregulated DAPs and 11 of the 26 downregulated DAPs to show sequence homology with proteins linked to virulence in several bacteria species, including those from the B. cereus group. Btk sequences with homology to the oligopeptide ABC transporter (OppA), superoxide dismutase (SOD), and polyketide synthase (PKS) were among upregulated virulence factors (Table 1). In contrast, Btk sequences with homology to flagellin (FlaA), internalin-like (InlA), and the nonhemolytic enterotoxin lytic component L1 (NheB) were downregulated (Table 2). Additionally, we identified an upregulated DAP (A0A1S7FFV1) with homology to an uncharacterized cytotoxin as a potential hemolymph-induced virulence factor in Btk.

**TABLE 1:** Upregulated putative virulence factors in hemolymph-treated Bacillus thuringiensis *kurstaki* cells predicted with the online tool VFDB

Accession	Gene name	BLASTP sequence homology^*a*^	E value
A0A2T7YM17	C5676_03879	PhoR: sensor histidine kinase (Mycobacterium smegmatis str. MC2 155) VFG009818	4E−20
Q3EKY9	RBTH_01584	PvdL: peptide synthase (Pseudomonas syringae *tomato* str. DC3000) VFG016061	6E−32
A0A2C1C3Q9	COJ15_35455	WcbT: 8-amino-7-oxononanoate synthase (capsular polysaccharide I) (Burkholderia thailandensis E264) VFG026073	3E−52
A0A2B4EE79	COJ78_05795	OppA: oligopeptide ABC transporter (oligopeptide-binding protein) (Listeria monocytogenes *4b* str. F2365) VFG006783	1E−125
A0A242XDX9	BG08_1843	Lpg0754: acetyltransferase (LPS) (Legionella pneumophila Philadelphia 1) VFG045302	3E−06
A0A0F6FTK0	BG08_3740	VipF: hypothetical protein (type IVB secretion effectors) (Legionella pneumophila str. Lens) VFG010772	4E−25
A0A2T7YRG6	C5676_02345	NarK2: nitrate/nitrite transporter (Mycobacterium *canettii* CIPT 140070017) VFG026850	1E−34
A0A243K4T0	BK741_25500	WcbT: 8-amino-7-oxononanoate synthase (capsular polysaccharide I) (Burkholderia thailandensis E264) VFG026073	3E−52
A0A243ITM2	BK708_25460	SodB: superoxide dismutase (Legionella pneumophila str. Philadelphia 1) VFG001867	7E−40
A0A347VE78	BG08_1971	Pks15/1: polyketide synthase (Mycobacterium liflandii 128FXT) VFG029280	3E−38

a VFDB, virulence factor database (http://www.mgc.ac.cn/VFs/; last accessed on 10 May 2021). VFDB contains 27,481 protein sequences related to known and predicted virulence factors of pathogenic bacteria (94).

**TABLE 2:** Downregulated putative virulence factors in hemolymph-treated Bacillus thuringiensis *kurstaki* cells predicted with the online tool VFDB

Accession	Gene name	BLASTP sequence homology^*a*^	E value
A0A2T0ESB8	C6351_29520	Lap: *Listeria* adhesion protein (Listeria seeligeri 1/2b str. SLCC3954) VFG031961	0.0
A0A137RUW0	AYK81_26425	FlaA: flagellin (Listeria monocytogenes EGD-e) VFG043258	5E−60
A0A243IT12	BK704_10345	DevR/S: response regulator (Mycobacterium gilvum Spyr1) VFG024199	7E−34
A0A347VBR2	CUC43_26340	Hmw2: cytoadherence accessory protein (Mycoplasma genitalium G37) VFG016449	1E−12
A0A2J9CTS8	CBR59_29605	InlA: internalin-like (Bacillus thuringiensis str. Al Hakam) VFG016359	7E−34
A0A1B1LDY3	xlyB_2	EntD: *N*-acetylmuramoyl-l-alanine amidase, family 2 (enterotoxin) (Clostridium perfringens ATCC 13124) VFG012148	2E−26
A0A243K1W7	BK741_29175	Hlp/LBP: histone-like protein/laminin-binding protein (Mycobacterium leprae TN) VFG043551	7E−17
J3QW49	NheB	NheB: nonhemolytic enterotoxin lytic component L1 (Bacillus thuringiensis *konkukian* str. 97-27) VFG016280	0.0
A0A2T7YV03	C5676_00021	IroE: salmochelin siderophore (Escherichia coli 536) VFG012502	1E−22
A0A242VXY1	BK699_33410	AcrA: acriflavine resistance protein A (Klebsiella pneumoniae NTUH-K2044) VFG049133	5E−04

a VFDB, virulence factor database (http://www.mgc.ac.cn/VFs/; last accessed on 10 May 2021). VFDB contains 27,481 protein sequences related to known and predicted virulence factors of pathogenic bacteria (94).

**FIG 4:**
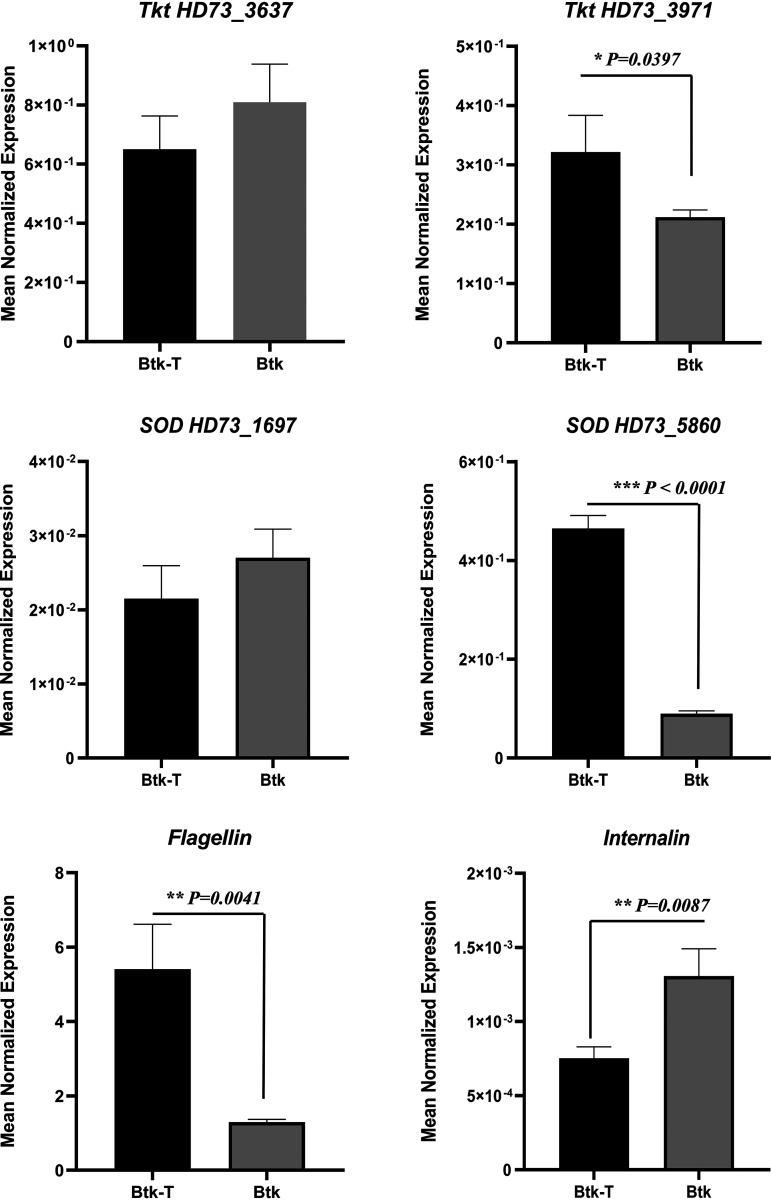
Transcriptional analysis of selected DAPs by two-step quantitative real-time PCR (qRT-PCR). The expression of Btk genes encoding transketolase, SOD, flagellin, and internalin DAPs was analyzed by qRT-PCR to verify changes in protein expression as determined by proteomic analysis. The detected transcript amounts were normalized to the reference gene *RpsU* with the aid of Q-gene software. Bars represent means of three independent replicates ± standard error. The Student’s *t* test (two tailed) was used to produce the *P* values shown in the graph.

Some Btk genes encoding DAPs (transketolase, SOD, flagellin, internalin) were selected for two-step quantitative real-time PCR (qRT-PCR) to verify changes in protein expression as determined by proteomic analysis. In the genome of Btk HD73 (GenBank accession no. CP004069), the sequences HD73_3637 (AGE79215) and HD73_3971 (AGE79549) encode transketolases (Tkt), HD73_1697 (AGE77275) and HD73_5860 (AGE81437) encode SODs, HD73_1893 encodes flagellin (AGE77471), and HD73_1560 encodes internalin (AGE77138). The qRT-PCR results showed that Btk hemolymph exposure significantly increased transcript levels of transketolase HD73_3971 (*t*[4] = 3.01, *P* < 0.0397) and SOD HD73_5860 (*t*[4] = 23.90, *P* < 0.0001) and decreased the expression of internalin (*t*[4] = 4.78, *P* < 0.0087), which was consistent with our proteomic data (Fig. 4). However, the mRNA levels of *flagellin*, previously identified among downregulated DAPs, were found to be significantly higher in Btk-T than in Btk cells (*t*[4] = 5.91, *P* < 0.0041). A limited correlation between gene transcription and protein abundance has previously been described (40–42).

### Hemolymph enhances Btk’s autoaggregation and biofilm formation.

**FIG 5:**
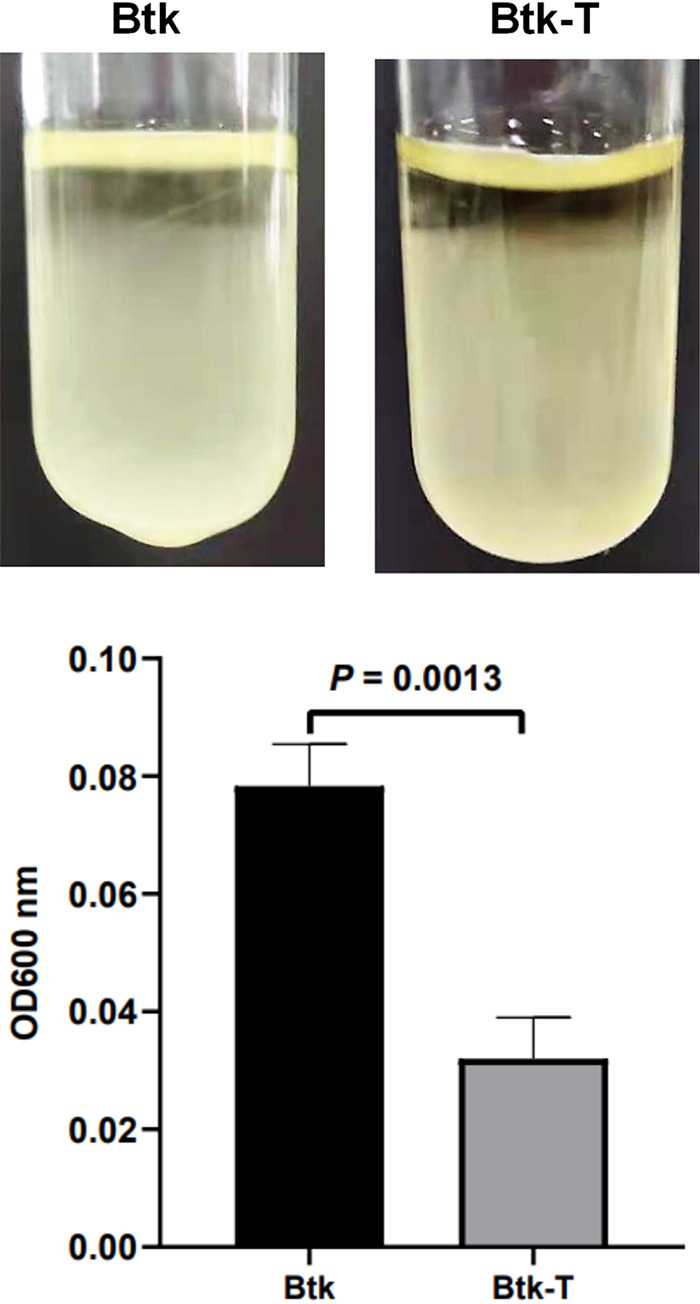
Effects of hemolymph exposure on Btk cell autoaggregation. The formation of multicellular aggregates was found to be enhanced in Btk-T (hemolymph-treated) versus Btk (control) cells during sedimentation assays. Top, representative images of the tubes after 16 h of static incubation at room temperature; bottom, OD_600_ values of cell suspensions at the air-liquid interface. Means were compared using a *t* test (*P < *0.05).

Autoaggregation is crucial for the virulence of pathogenic bacteria because of the multiple advantages it can provide, such as increasing the tolerance against antimicrobials (43–45), blocking phagocytosis by host immune cells (46), and improving survival within phagosomes (47), that prolong bacterial persistence within the host. We hypothesized that hemolymph stimulation would enhance the formation of Btk aggregates in static liquid cultures. To address this issue, we performed sedimentation assays in Btk-T and Btk by measuring the optical density at 600 nm (OD_600_) of liquid cultures from the top of the tubes after 16 h of static incubation at room temperature. The results showed that OD values significantly decreased in Btk-T compared with control Btk (*t*[4] = 8.052, *P* = 0.0013), which indicated that the formation of Btk aggregates is enhanced by hemolymph (Fig. 5).

**FIG 6:**
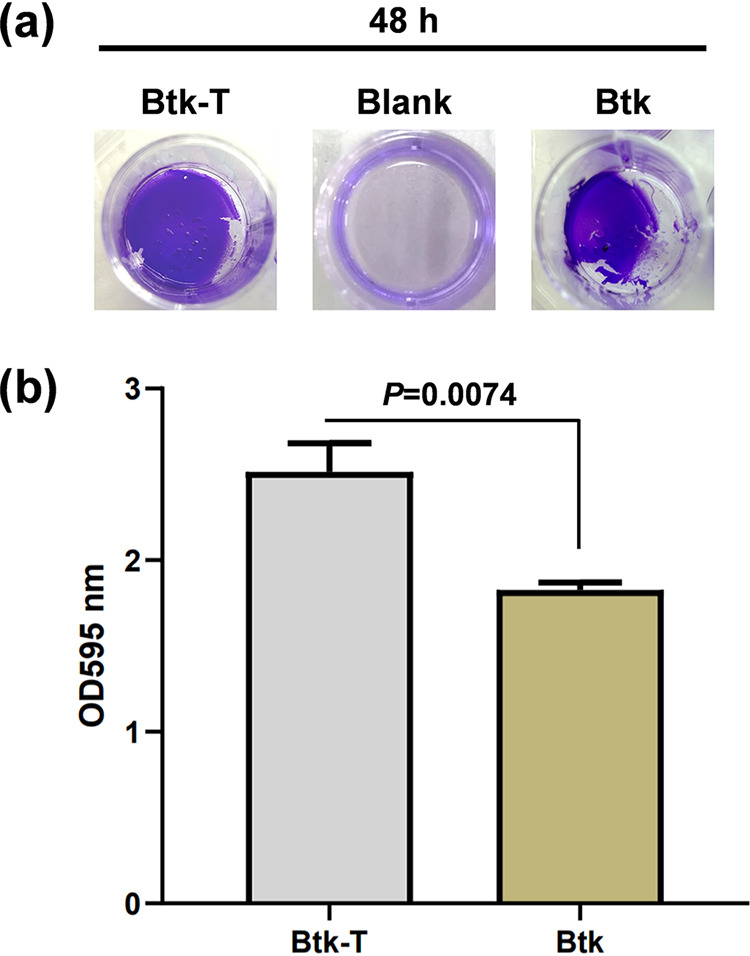
Effects of hemolymph exposure on biofilm formation by Btk. A crystal violet assay showed 48-h-old biofilm levels produced by Btk significantly increased after hemolymph treatment. (a) Visualization of crystal violet-stained 48-h-old biofilms formed by Btk-T and Btk cells at the bottom of individual wells in a 24-well plate compared to a medium blank control. (b) Graphical representation of crystal violet quantification of biofilm formation in Btk-T and Btk. The crystal violet stain was washed out from biofilms with 95% ethanol and measured as optical density readings (OD_595_). Bars represent means of three independent replicates ± SE. A Student’s *t* test (two tail) was used to produce the *P* value shown in the graph.

Cell aggregation is an early step in the formation and maturation of bacterial biofilms. Therefore, we investigated the effects of hemolymph on Btk’s biofilm formation on microtiter plates. Crystal violet (CV) staining visualized 48-h-old bacterial biofilms attached to well bottoms in a 24-well plate, which were more expanded on the surface and contained more multilayer cell clusters (microcolonies) for Btk-T than for Btk (Fig. 6a). Biofilm quantification (OD_595_) by ethanol extraction of crystal violet confirmed that biofilm levels produced by Btk significantly increased after hemolymph treatment (*t*[3] = 6.49, *P* = 0.0074) (Fig. 6b).

### Hemolymph induces epitope masking of Btk’s selective surface proteins.

**FIG 7:**
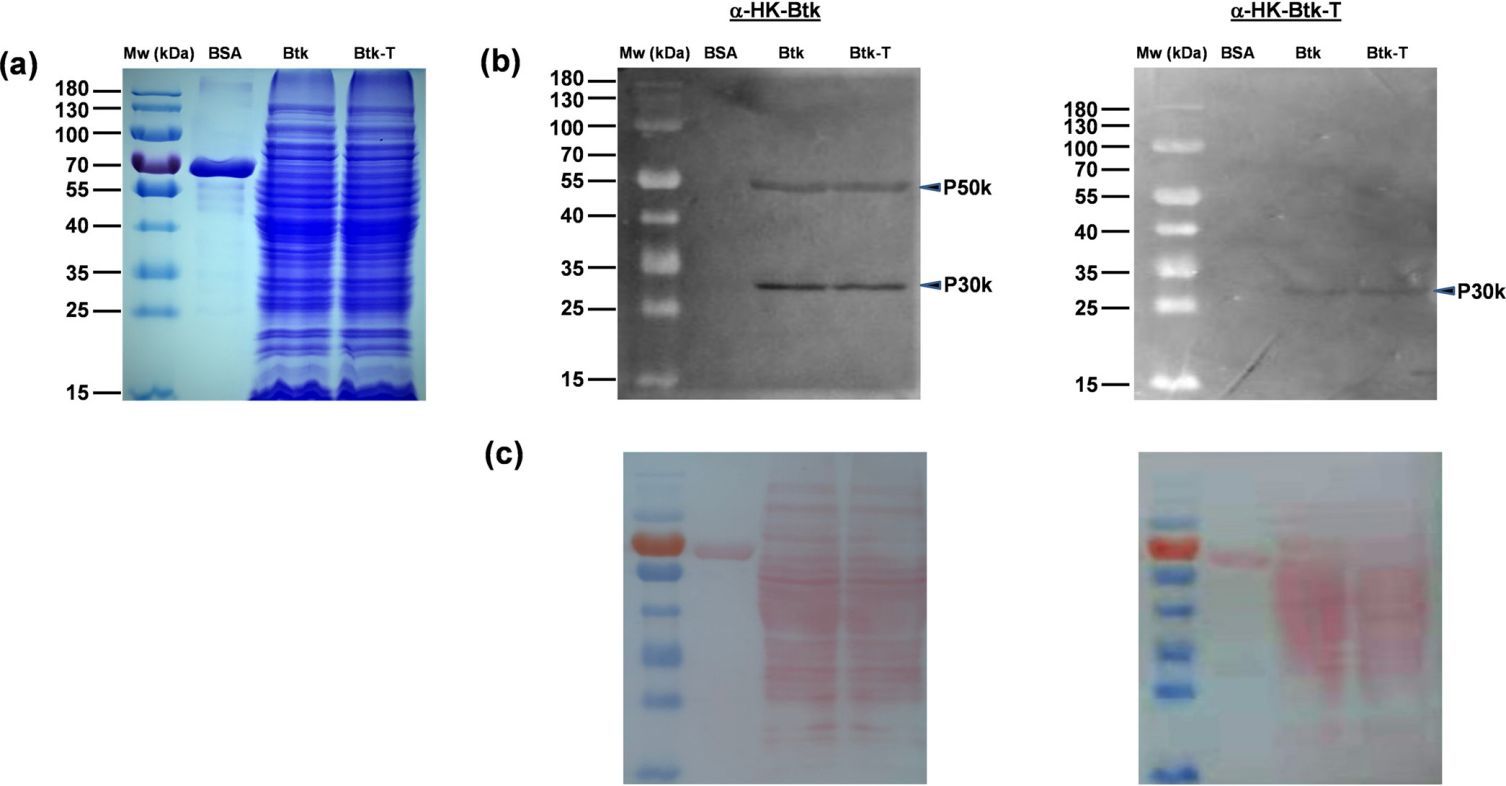
Immunological characterization of Btk and Btk-T cell surface proteins. Western blotting experiments of Btk and Btk-T surface protein extracts using antisera raised in BALB/c mice against heat-killed Btk (anti-HK-Btk) or Btk-T (anti-HK-Btk-T) cells revealed that hemolymph exposure by Btk decreased the bacterium immunogenicity in terms of the detection of two major antigens in the Btk surfaceome (i.e., P30k and P50k by the mouse immune system). (a) Twenty micrograms of Btk and Btk-T protein from the insoluble fraction of bacterial lysates, comprised mostly of proteins from the plasma membrane and cell wall, was loaded into 10% SDS-PAGE under reducing conditions; BSA, bovine serum albumin (1 μg); Mw, molecular weight (kDa), PageRuler (15 to 180 kDa) prestained protein marker (Thermo Scientific). (b) Electrophoresed proteins were blotted onto nitrocellulose membranes and then probed with mouse antiserum raised against heat-killed Btk (left) or heat-killed Btk-T (right) immunogens. The working dilution of mouse antisera was 1:100 in TBST (50 mM Tris, pH 7.6, 150 mM NaCl, 0.2% Tween 20). A commercial goat anti-mouse IgG-HRP-conjugated antibody (1:10,000 in TBST) was used for detection. Blots were developed using the Thermo Scientific Pierce ECL Western blotting substrate. Arrows indicate the P30k and P50k antigens of Btk and Btk-T. (c) Ponceau S (0.1%) reversible staining of the blots.

To successfully colonize the insect host, hemolymph exposure by Btk could trigger different strategies in the bacterium to hide PAMPs from recognition by the insect immune system and avoid clearance. To test this assumption, we investigated whether the immunogenicity of Btk’s cell surface proteins changed after hemolymph treatment (i.e., Btk-T). We immunized BALB/c mice with heat-killed (HK) Btk cells (HK-Btk) or HK Btk-T cells (HK-Btk-T) and compared both antisera in Western blotting experiments of Btk and Btk-T surface proteins (Fig. 7a, b, and c). The HK-Btk mouse antiserum immunoreacted with two bands of ∼30 and ∼50 kDa, designated P30k and P50k, respectively, present at a similar concentration in Btk and Btk-T surface protein extracts (Fig. 7b, left). However, the HK-Btk-T mouse antiserum showed lower anti-P30k antibody titers and failed to detect the P50k antigen in the Western blot of the same Btk and Btk-T surface protein extracts (Fig. 7b, right), suggesting that Btk-T was less visible than Btk to the mouse immune system.

### Hemolymph preexposure increases Btk toxicity to injected insects.

To investigate the effects of hemolymph treatment on Btk toxicity, we injected fifth instar *Bo. mori* larvae intrahemocoelically with various doses of Btk-T and Btk (3, 20, 70, and 500 bacteria/injected larvae) and recorded the number of dead larvae after 24 h. In these bioassays, Btk-T cells were significantly (*P < *0.01) more toxic (ca. 2.4-fold; 50% lethal concentration [LC_50_] = 2.96 ± 0.79 bacteria/injected larva; 95% fiducial limits (FL) 1.41 to 4.51; slope ± standard error [SE] = 2.05 ± 0.17) than control Btk cells (LC_50_ = 6.99 ± 0.50 bacteria/injected larva; 95% fiducial limits 6.01 to 7.97; slope ± SE = 2.48 ± 0.18).

**FIG 8:**
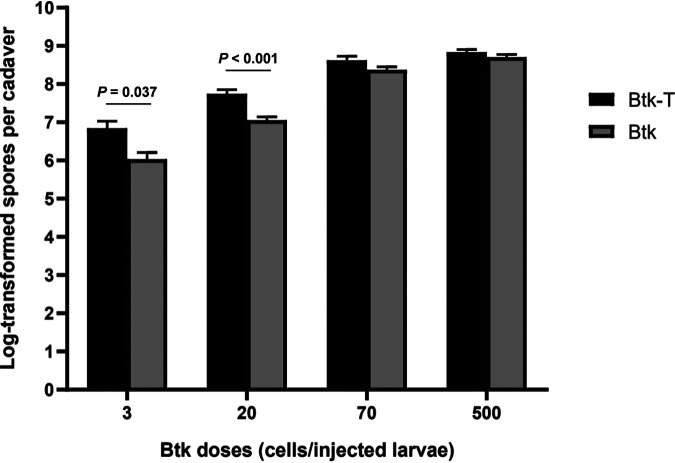
Effects of hemolymph exposure on the relationship between Btk reproduction within the insect and bacterial dose in the inoculum. The reproduction of Btk-T and Btk was dose dependent and significantly higher for Btk-T at low bacterial doses. Data are log-transformed spore counts per cadaver. Bars represent means ± SE. A Student’s *t* test (two tailed) was used to produce the *P* values shown in the graph.

The virulence of Btk-T and Btk was further compared by quantifying bacterial reproduction as spore counts per cadaver in the different concentrations of inoculum. We found that reproduction of Btk-T and Btk increased with the dose, but it was significantly higher for Btk-T at low concentrations of the inoculum, that is, 3 (*t*[10] = 3.04, *P* = 0.037) and 20 (*t*[15] = 4.86, *P* < 0.001) bacteria/injected larvae (Fig. 8).

## DISCUSSION

In this study, we used TMT-based quantitative proteomics to identify a set of Btk proteins whose synthesis rate changed in response to *Bo. mori* hemolymph and have called it the Btk hemolymph “stimulon.” The GO classifications showed that DAPs were enriched mainly in glutamate metabolism, transketolase activity, and transmembrane transport. Our experimental design was not intended to investigate Btk proteome alterations during bacterial cell multiplication within the insect blood but rather alterations occurring after exposure of bacterial cells to small quantities of hemolymph, which simulates the early stages of invasion when the first bacterial cells have crossed the midgut epithelial barrier. Several PlcR-regulated extracellular virulence factors have previously been shown to facilitate such passage, including an enhancin-like Bel protease involved in the hydrolysis of the invertebrate intestinal mucin in the peritrophic membrane (48), a semipermeable chitin-rich structure lining the midgut of most insects (49), and a ColB collagenase that digests cadherins and the basal lamina underlining the midgut epithelium (50, 51). However, the only PlcR-regulated gene product reported in the Btk hemolymph stimulon, the nonhemolytic enterotoxin NheB (16), was downregulated, suggesting that the PlcR virulence regulon is not required for the response to hemolymph.

Heterogeneity in bacterial populations has been described as an adaptive trait to environmental insults, where the activation of different lifestyles in genetically identical cells is a result of the interaction between external and internal factors (52–54). Verplaetse et al. described different Bt-specialized subpopulations coexisting throughout the insect colonization process (13). These authors classified infesting Bt cells into four different physiological stages, including virulence, necrotrophism, sporulation, or undefined depending on whether PlcR, NprR, Spo0A, or an as-of-yet unknown regulator was activated, respectively. Ben Rejeb et al. found that about 20% of the Bt population in insect cadavers was undefined and suggested classifying these cells as a new dormant subpopulation because most of them were alive but not growing (19). Neither PlcR nor NprR nor Spo0A regulons were activated in the Btk hemolymph stimulon, suggesting that these cells could represent a new specialized subpopulation. However, we identified an upregulated DAP sequence (A0A0G4CXH7) with homology to the CarD-like family of transcriptional regulators required in Mycobacterium tuberculosis survival to oxidative stress and persistence (55). We also found an upregulated DAP (A0A0B5NQ68) with homology to the phosphate-specific transport system accessory protein PhoU involved in the formation of persister cells. Persisters are transient (nongrowing or slow-growing) phenotypic variants of a clonal bacterial population that show resistance to multiple adverse conditions, such as antimicrobials and oxidative stress (56, 57). Persistence can play essential roles during bacterial pathogenesis. For instance, pathogenic persisters of Salmonella enterica serovar *Typhimurium* and M. tuberculosis have been found to induce *in vivo* macrophage bactericidal activity suppression, creating a more favorable environment for infection in the host (58, 59). The formation of persisters in Bt vegetative cells has yet to be identified; however, this phenomenon is widely spread among prokaryotic microorganisms, including other Gram-positive *Firmicutes* Staphylococcus aureus and Bacillus subtilis (60, 61). A potential outcome of hemolymph exposure by Btk could involve forming a small subpopulation of persisters to deal with host immune reactions, such as the oxidative burst of macrophages while weakening the host defenses using unrevealed effectors. Further molecular studies are needed to fully characterize the Btk phenotypic variant expressing a hemolymph stimulon.

Two upregulated DAPs identified as transketolase were enriched in the “biosynthesis of ansamycins” pathway, suggesting that these bacterial macrocyclic polyketides with demonstrated antimicrobial (62), anticancer (63), and antiviral (64) activities could be playing important roles in Btk’s survival in the hemolymph. The only known ansamycins of *Bacillus* origin are the antibiotic aurantinin and antivirals hydroxymycotrienins A and B (65, 66). Transketolases catalyze the production of 1-deoxy-1-imino-d-erythrose 4-phosphate (iminoerythrose-4P), the substrate for the formation of 4-amino-3,4-dideoxy-d-arabino-heptulosonic acid 7-phosphate (aminoDAHP), the first intermediate of the aminoshikimate pathway (67, 68). The final product of the aminoshikimate pathway is 3-amino-5-hydroxybenzoic acid (AHBA), which in turn is the precursor of ansamycin biosynthesis (69). However, transketolase is also a key enzyme of the oxidative branch of the pentose phosphate pathway, which contributes to bacterial tolerance (persistence) to oxidative stress by producing the cellular NADPH required for intracellular reactive oxygen species detoxification (70). In St. aureus, the inactivation of the transketolase-encoding *tkt* gene affected bacterial pathogenesis, particularly the intracellular proliferation and tissue-colonizing ability (71). Whether upregulation of transketolases in hemolymph-responsive bacteria is related to the production of ansamycins or to link central metabolism to redox homeostasis needs further investigation.

Several well-known virulence factors in other bacterial species were found to be differentially expressed in the Btk hemolymph stimulon. A sequence with homology to SOD was among upregulated DAPs in hemolymph-exposed Btk cells. SOD is part of all organisms’ antioxidant defense systems and catalyzes the dismutation reaction of superoxide anion (O_2_^−^) to molecular oxygen and hydrogen peroxide (H_2_O_2_); peroxidases and catalases then reduce the H_2_O_2_ into H_2_O and O_2_. Pathogens produce SOD to survive the superoxide stress generated by host phagocytic cells, a conserved response of innate immunity (72, 73). Further analysis by qRT-PCR of two SOD-encoding sequences in the Btk HD73 genome (i.e., HD73_1697 and HD73_5860) only showed significant expression changes due to hemolymph for the latter, which has homology to manganese-SOD (MnSOD)-like genes. Previously, Coburn et al. found a Ba. cereus strain causing human endophthalmitis where MnSOD-encoding gene variants *sodA1* and *sodA2* were among the most highly expressed bacterial genes in *ex vivo* vitreous humor (74).

In addition to NheB, two other experimentally verified Bt virulence factors, flagellin and internalin, were identified among downregulated DAPs in the Btk hemolymph stimulon, suggesting that their participation is not required for bacterial survival and replication within living insect hemolymph but is in other stages of pathogenesis. Flagellin is the major structural protein of bacterial flagellum and is involved in host cell adherence, biofilm formation, protein secretion, and host cell invasion (75). Zhang et al. showed that a nonmotile flagellin mutant of Bt *gelechiae* was less virulent when fed to *Trichoplusia ni* but not when injected into the larval hemocoel (76), implying a major role of flagellin following oral infection. The downregulation of flagellin in the Btk hemolymph stimulon can also reflect a survival strategy to escape the circulating host innate immune sensing considering that this bacterial protein is at the top of the immunodominance hierarchy (77). Similarly, the expression of a Ba. cereus internalin-like protein, named IlsA for iron-regulated leucine-rich surface protein previously identified through an *in vivo* expression technology approach (78), was undetected in bacteria recovered from infected Galleria mellonella larvae when the insects were still alive but was very strong in those from insect cadavers (79).

In our study, Btk cells exposed to hemolymph formed more aggregates in liquid culture and biofilm pellicles attached to a submerged solid surface (i.e., the wells of microtiter polystyrene plates). Biofilms are complex communities of sessile bacteria that secrete extracellular polymeric substances for a firm attachment to surfaces and protection from host immunity, including phagocytosis (80–82). The exposure of microbial pathogens to damaged tissues is critical for biofilm infections (83). Interestingly, neither TasA nor CalY, two extracellular proteins involved in the production of biofilm matrix fibers in members of the Ba. cereus group (38, 84), were identified in the Btk hemolymph stimulon, suggesting that increased biofilm formation was a consequence of enhanced autoaggregation followed by sedimentation in treated over control bacteria. Bacterial autoaggregation has been found to prevent phagocytosis by the host macrophages (46, 85). It can also protect bacteria against antimicrobials and oxidative stress generated by host immune cells (43–45). Neutrophil exposure by Pseudomonas aeruginosa, the causal pathogen of cystic fibrosis lung disease, induced the formation of bacterial aggregates and biofilms that conferred antibiotic resistance and enhanced virulence (45, 86). For Campylobacter spp., autoaggregation occurs after adhesion to host target cells, which could modulate virulence by increasing the concentration of flagella-secreted virulence factors during the pathogenic process (87). The formation of multicellular aggregates could also be advantageous for Btk survival in the hemolymph to confer protection from insect cellular and humoral defenses while enhancing its invasion and colonization.

Successful pathogens use anti-immune strategies to avoid host clearance and cause disease (88–91). We found that hemolymph exposure by Btk changed the immunogenicity *in vivo* of bacterial surface proteins verified through immunization experiments in mice. Two major Btk surface antigens, P30k and P50k, were more or less immunoreactive whether Btk or Btk-T was used as the immunogen. The explanation for this observation could be that interaction with hemolymph triggers evasion mechanisms in Btk, such as PAMP masking, to make the pathogen invisible to insect immune defenses while causing bacteremia in the site of infection. In bioassays with intrahemocoelically infected insects, Btk-T was more virulent and showed a higher reproduction rate within the hemocoel at low inoculum doses than Btk, suggesting that the former can avoid phagocytosis and other mechanisms of antibacterial killing.

In summary, we have shown that Btk triggers a hemolymph stimulon following contact with insect hemolymph, which could be partially responsible for bacterial survival and colonization of the insect body. Insect hemolymph propagation and septicemia induction by Bt are clearly complex; however, it remains uncertain whether or not the DAPs identified in this study are directly involved in these processes. Future experiments in our laboratory with many of these DAPs will try to determine their exact role in Btk pathogenesis through gene knockout or genome editing in the bacterium. Finally, our findings can contribute to the understanding of the mechanisms used by Bt to overcome insect defenses and kill the host that could also be used by other members of the Ba. cereus group, such as Ba. cereus and Ba. anthracis, which are important human pathogens.

## MATERIALS AND METHODS

### Insects and Bt strain.

A population of *Bo. mori* strain Dazao p50, obtained from the Sericultural Research Institute, Chinese Academy of Agricultural Sciences, was raised on leaves of white mulberry (*Morus alba*) at the Insect facility of the Biology Department at the Nanyang Normal University (Nanyang, Henan, People’s Republic of China). The *Bo. mori*-rearing conditions were 25°C ± 2°C, ca. 60% relative humidity, and a 12-h light/12-h dark cycle. Insect hemolymph was collected from a droplet squeezed from an abdominal incision in early fifth (last) larval instars.

Ba. thuringiensis *kurstaki* strain HD73 (Btk) catalogue no. BGSC-4D4 of the Bacillus Genetic Stock Centre (Columbus, Ohio) was kindly gifted by Daniel R. Zeigler and preserved in our lab as bacterial spores on filter disks at 4°C.

### Bacterial exposure to insect hemolymph.

A filter disk containing Btk spores was hydrated with Luria broth (LB) and inoculated onto LB agar plates overnight at 30°C to revive the culture. Cells were streaked on fresh LB agar plates for isolation of single colonies.

For Btk liquid cultures, a 10-ml LB culture was inoculated with a single colony and grown with vigorous shaking (220 rpm) overnight at 30°C to reach saturation. This culture was then used to inoculate fresh 50 ml of LB (1:100, vol/vol) in a 250-ml flask and allowed to grow until the OD_600_ reached approximately 0.4 to 0.6 (mid-log) when it was divided into two 25-ml cultures in 100-ml flasks. *Bo. mori* hemolymph was added to one of the cultures (1:100, vol/vol). Following 60 min of static incubation at room temperature, hemolymph-treated (Btk-T) and untreated (Btk) cultures were centrifuged (3,000 × *g*, 10 min, 4°C), and resultant cell pellets were washed twice with 1× phosphate-buffered saline (PBS) buffer (137 mM NaCl, 10 mM Na_2_HPO_4_, 1.8 mM KH_2_PO_4_, 2.7 mM KCl, pH 7.4) and stored frozen at −80°C for further use.

### Protein extraction, digestion, and TMT labeling.

Btk-T and Btk pellets were resuspended in SDT buffer (4% SDS, 100 mM Tris-HCl, 1 mM DTT, pH 7.6) for bacterial lysis and protein extraction. Protein concentration was determined with a detergent-compatible formulation based on bicinchoninic acid (BCA) in the Pierce BCA protein assay kit (Thermo Scientific), using bovine serum albumin (BSA) as the standard.

Trypsin digestion of proteins (trypsin/protein mass ratio 1:50 overnight at 37°C) was performed according to filter-aided sample preparation (FASP) procedure (92). Digested peptides were desalted on C_18_ cartridges (Empore SPE Cartridges, Sigma), freeze dried, reconstituted in 0.1% (vol/vol) formic acid, and quantified in a spectrophotometer (OD_280_).

The tryptic peptides (100 μg of protein) were categorized to label with 126-tag (Btk1), 127-tag (Btk2), 128-tag (Btk3), 129-tag (Btk-T1), 130-tag (Btk-T2), and 131-tag (Btk-T3) using a TMT labeling kit (Thermo Fisher Scientific) according to the manufacturer’s instructions.

### Fractionation and liquid chromatography-tandem mass spectrometry analysis.

Liquid chromatography-tandem mass spectrometry (LC-MS/MS) analysis on a Q Exactive mass spectrometer (Thermo Scientific) coupled to an Easy nLC (Thermo Fisher Scientific) was performed by the Shanghai Applied Protein Technology Co., Ltd. (Shanghai, China). The peptides were loaded onto a reverse-phase trap column (Thermo Scientific Acclaim PepMap100, 100 μm × 2 cm, nanoViper C_18_) connected to the C_18_ reversed-phase analytical column (Thermo Scientific Easy column, 10-cm long, 75-μm inner diameter, 3-μm resin) in buffer A (0.1% formic acid) and separated with a linear gradient of buffer B (84% acetonitrile and 0.1% formic acid) at a flow rate of 300 nl/min controlled by IntelliFlow technology. The mass spectrometer was operated in positive ion mode. MS data were acquired using a data-dependent top10 method dynamically choosing the most abundant precursor ions from the survey scan (300 to 1800 *m*/*z*) for high-energy collisional dissociation (HCD) fragmentation. The automatic gain control (AGC) target was set to 3E6 and maximum inject time to 10 ms. Dynamic exclusion duration was 40.0 s. Survey scans were acquired at a resolution of 70,000 at *m*/*z* 200, the resolution for HCD spectra was set to 17,500 at *m*/*z* 200, and isolation width was 2 *m*/*z*. Normalized collision energy was 30 eV, and the underfill ratio, which specifies the minimum percentage of the target value likely to be reached at maximum fill time, was defined as 0.1%. The instrument was run with peptide recognition mode enabled.

### Protein identification and quantitative analysis.

The MS raw data for each sample were searched using the MASCOT engine (Matrix Science, London, UK; version 2.2) embedded into Proteome Discoverer 1.4 software for identification and quantitation analysis. The following search parameters were used: missed cleavages, 2; peptide mass tolerance, ±20 ppm; fragment mass tolerance, 0.1 Da; fixed modifications, carbamidomethyl (C), TMT 6-plex; variable modifications, oxidation (M); database, uniprot_Bacillus_thuringiensis_466681_20191013.fasta; protein false discovery rate (FDR) threshold, ≤0.01. All contaminant and decoy proteins were removed from the data sets before downstream analysis. The protein ratios were calculated as the median of the protein’s unique peptides and normalized by taking the median of all calculated proteins. Unpaired two-tailed Student’s *t* tests were performed to analyze the expression differences of proteins from Btk-T and Btk (*P < *0.05). The FDR was used to calculate the *P* value in the significance tests, and resulting *P* values were adjusted using Benjamini and Hochberg’s method (93).

### Bioinformatic analysis.

Homologue sequences of identified proteins were retrieved using the NCBI BLAST+ client software (ncbi-blast-2.2.28+-win32.exe). Then, GO terms were mapped, and sequences were annotated using Blast2GO. The prediction of subcellular localization was done through CELLO (http://cello.life.nctu.edu.tw/), and domain functional descriptions were annotated by the InterPro database (https://www.ebi.ac.uk/interpro/search/sequence/). Proteins with enzymatic functions, possibly involved in metabolic pathways, were predicted by the KEGG pathway database (http://www.genome.jp/kegg/pathway.html). GO enrichment on three ontologies (biological process, molecular function, and cellular component) and KEGG pathway enrichment analyses were applied based on two-sided Fisher’s exact tests, considering the whole quantified protein annotations as a background data set. Benjamini-Hochberg correction for multiple testing was further used to adjust derived *P* values.

Putative virulence proteins were *in silico* predicted by analyses for protein sequence similarity to virulence factors of pathogenic bacteria using the online tool VFDB (virulence factor database; http://www.mgc.ac.cn/VFs/) (94) and BLASTP homology searches (95). A cutoff for significant sequence homology was set at an E value less than 10^−4^ to indicate homology between Btk sequences and bacterial sequences.

### RNA isolation, cDNA synthesis, and two-step quantitative real-time PCR.

Total RNA from Btk-T and Btk pellets was isolated with the Easy Pure RNA kit (TransGen Bioech). Multiple DNase I-treated RNA preparations for each treatment were pooled into hemolymph-treated (Btk-T) and control (Btk) groups before ethanol precipitation. The RNA quality was verified by 1% agarose gel electrophoresis stained with GelRed (Biotium). The RNA concentration was measured using a NanoDrop 2000 spectrophotometer (Thermo Scientific). Btk-T and Btk total RNA (2 μg) samples were annealed with 25 ng of random hexamer primers and used for cDNA synthesis with the aid of a RevertAid first strand cDNA synthesis kit (Thermo Fisher, USA). Negative controls lacking the reverse transcriptase were prepared to verify the absence of contaminating genomic DNA.

Two-step qRT-PCRs were run on a Bio-Rad CFX96 Touch real-time PCR system (Bio-Rad) using Roche FastStart universal SYBR green master mix (Rox) (Roche) according to the manufacturer’s protocol. The 25-μl SYBR green PCRs were performed in triplicate with 100 ng of template cDNA and 7.5 pmol of each primer (Table S7 in the supplemental material; primers were designed according to Primer 3 online software at http://primer3plus.com/cgi-bin/dev/primer3plus.cgi). A 102-bp amplicon from Btk 30S ribosomal protein S21 (*rpsU*) gene (GenBank accession no. CP004069) was used for transcript normalization (96). The PCR cycling conditions were as follows: an initial denaturation at 95°C for 3 min followed by 40 cycles of denaturation at 95°C for 15 s, annealing at 60°C for 30 s, and extension at 72°C for 30 s. Specificity of the PCRs was confirmed by melting curve analysis at 55 to 95°C, and all threshold cycle (*C_T_*) values were obtained from three independent experiments. Amplification efficiencies of both target and reference genes were determined with the aid of standard curves (Table S7). The relative transcript levels were expressed as “Mean normalized Expression” data using Q-GENE software (http://www.gene-quantification.de/qgene.zip).

### Sedimentation assay.

Bacterial autoaggregation was determined by a sedimentation assay (97) using the final point measurement method. Ten milliliters of the 60-min static Btk-T or Btk cultures was added into narrow culture tubes and incubated vertically without agitation for 16 h at room temperature. The sedimentation of cell aggregates was then determined in terms of culture turbidity from the top of the tubes. Ten microliters of the medium was carefully taken from the air-liquid interface and diluted in 90 μl of LB, and the absorbance was determined at OD_600_ in a BioPhotometer (Eppendorf).

### Biofilm formation assays.

Changes in biofilm formation due to hemolymph exposure were assayed by the ability of Bt cells to adhere to the wells of 24-well polystyrene plates (98). LB medium was preequilibrated for 16 h at 30°C in 24-well polystyrene plates (Falcon) before inoculation with 1:10 dilutions of 60-min static Btk-T or Btk cultures. Plates were produced in triplicate, and each plate contained three wells per treatment and control (blank) LB medium. Plates were sealed with a plastic film to prevent evaporation and bacteria grown statically for 48 h at 30°C. Medium containing planktonic cells were then removed by pipetting, after which the wells were washed once with 700 μl of 1× PBS and incubated for 30 min at room temperature with 700 μl of filter-sterilized 1% (vol/vol) crystal violet (CV) solution (Solarbio). The excess of CV was removed from the wells, followed by three washes with 1× PBS. For quantitation of biofilm formation, CV was extracted by adding 1 ml of 95% ethanol to each well and incubated for 15 min at room temperature. The CV/ethanol solution was then transferred to a plastic cuvette, and absorbance was determined at 595 nm in an Ultrospec 3000 spectrophotometer (Amersham Biosciences).

### Mice immunization.

Immunization of mice was performed in strict accordance with the recommendations of the Ethics Committee of Experimental and Animal Welfare of the Chinese Association for Laboratory Animals (99). The protocol was approved by the Animal Care and Use Committee at the Nanyang Normal University. All animals were anesthetized with diethyl ether and euthanized by cervical dislocation to minimize animal suffering.

To prepare the inoculum, Btk-T and Btk pellets were resuspended into 1× PBS, adjusted to 1 × 10^8^ bacteria per ml with the aid of a hemocytometer, and heat killed (HK) at 70°C for 30 min in a dry block heater. Nonviability of heat-treated suspensions was checked by spreading 0.1 ml of each suspension on LB plates. The HK-Btk-T and HK-Btk immunogens thus prepared and containing no adjuvant were stored at −80°C as 0.2-ml aliquots until use.

Six-week-old female BALB/c mice (Zhengzhou Fengmao Experimental Animal Co. Ltd., Henan, People’s Republic of China) were inoculated intraperitoneally with 0.2 ml of HK-Btk-T or HK-Btk immunogens (two mice each) four times 1 week apart. Two nonimmunized mice, used as controls, were maintained under similar conditions. Antisera were obtained at the terminal stage of the study by collecting the blood through cardiac puncture in anesthetized mice. The blood samples were centrifuged (3,000 × *g*, 4°C, 30 min), and the supernatants (sera) were collected, transferred to a clean tube, and stored at −20°C until use.

### SDS-PAGE and Western blotting.

Btk-T and Btk pellets were resuspended into 1× Tris-EDTA (TE) buffer (10 mM Tris, 1 mM EDTA, pH 8) containing lysozyme (3 mg/ml) and bacteria lysed by 450 sonication pulses (400 W, 3 s with a 5-s interval) cooled in an ice water bath. Suspensions were centrifuged (13,000 × *g*, 30 min, 4°C), and insoluble fractions were homogenized in buffer (0.3 M Tris-HCl, pH 6.8, 0.3 M NaCl, 4% SDS, and 0.1 M DTT) and boiled for 5 min to extract cell surface proteins. Protein concentration in the supernatants was determined with a BCA protein assay kit (Thermo Scientific) as above.

Protein samples (20 μg) were boiled for 5 min with 5× SDS sample buffer (0.3 M Tris-HCl, pH 6.8, 50% glycerol, 10% SDS, 0.125% bromophenol blue) supplemented with 5% β-mercaptoethanol for separation by reducing 10% SDS-PAGE (100). One gel was stained with Coomassie brilliant blue for protein visualization, while the other two gels were blotted onto nitrocellulose membranes using a semidry transfer device (Bio-Rad). Proteins in the blots were visualized with 0.1% Ponceau S reversible staining to determine transfer efficiency. Western blotting was performed as described previously (101) by adding 1:100 of primary antibody (mouse serum raised against HK-Btk-T or HK-Btk) and 1:10,000 of secondary antibody (goat anti-mouse IgG-horseradish peroxidase [HRP] conjugated; Thermo Fisher Scientific). Tris-buffered saline with Tween 20 (TBST) buffer (50 mM Tris, pH 7.6, 150 mM NaCl, 0.2% Tween 20) was used to dilute the antibodies and wash the blots. All Western blots were developed using the Thermo Scientific Pierce ECL Western blotting substrate (Thermo Fisher Scientific, Pierce) following manufacturer’s instructions.

### Pathogenicity in insects.

Btk-T and Btk pellets were resuspended into 1× PBS, counted with the aid of a hemocytometer, and used to prepare four infectious doses ranging from 10^1^ to 10^4^ cells/ml. Infectious doses were confirmed by CFU count after plating appropriate dilutions on LB agar (102). Groups of 12 early fifth instar larvae of *Bo. mori* ca. 5 cm in length and 1.5 g of weight were injected with bacterial suspensions (5 μl) into the base of the last proleg with a sterile Hamilton syringe (Sigma-Aldrich) via a 26 G × 1/2 in. (0.45 × 12 mm) needle. A control group of larvae was injected with 1× PBS alone. Insect mortality was scored after 24 h; a larva was considered dead if no movement was detected after being stimulated with a blunt-ended tip.

Reproduction of Btk-T and Btk within the insect was determined as spore counts per cadaver for the different bioassay doses. Dead larvae were individually crushed and homogenized in 1 ml of 1× PBS using a sterile glass pestle, and homogenates were heat treated at 70°C for 30 min to kill all vegetative bacteria. Serial dilutions of the homogenates were plated on LB agar, and colonies were counted after incubation at 30°C for 24 h (102). Homogenates produced from noninfected, living larvae (1× PBS-injected group) were used as negative controls.

### Data analysis.

Data were analyzed using GraphPad Prism software version 8.0.2 for Windows. Unpaired two-tailed Student’s *t* tests were used to determine differences between Btk-T and Btk cells regarding qRT-PCR mean normalized expression, autoaggregation, biofilm formation, and spore counts per cadaver. In all cases, a *P *value <0.05 was considered statistically significant.

For insect bioassays, the analysis was performed in R (http://www.R-project.org/). Estimates of LC_50_ (lethal concentration that kills 50% of the population) and their 95% fiducial limits (FL) were calculated by maximum likelihood logit regression analysis in a generalized linear model from individually fitted analyses of deviance. Pairwise comparisons of LC_50_ values were significantly different (*P < *0.01) if their respective 95% FL did not overlap.

The experiments were performed three times (on separate days) with three independent replicates per treatment, and similar results were obtained. The standard error of means was used to compare the replicates.

### Data availability.

The mass spectrometry proteomics data have been deposited to the ProteomeXchange Consortium via the PRIDE (103) partner repository with the data set identifier PXD021830.
