# Supplementary material for: Tandem Mass Tag-Based Quantitative Proteomics and Virulence Phenotype of Hemolymph-Treated Bacillus thuringiensis kurstaki Cells Reveal New Insights on Bacterial Pathogenesis in Insects
Source: Microbiol Spectr. 2021 Oct 27;9(2):e00604-21. doi: 10.1128/Spectrum.00604-21 (PMC8549738; doi:10.1128/Spectrum.00604-21)
Supplement: SUPPLEMENTAL FILE 1 — Fig. S1. Download Spectrum.00604-21-s0001.pdf, PDF file, 0.2 MB [file spectrum.00604-21-s0001.pdf]

Tandem mass tag-based quantitative proteomics and virulence phenotype of haemolymph-treated *Bacillus thuringiensis kurstaki* cells reveal new insights on bacterial pathogenesis in insects

Yanyan Sun[1,†], Linlin Yang[1,†], Lianet Rodríguez-Cabrera[2], Yushan Ding[1], Chaoliang Leng[1], Huili Qiao[1], Siliang Huang[1], Yunchao Kan[1], Lunguang Yao[1], Denis J. Wright[3], Dandan Li[1,*] and Camilo Ayra-Pardo[1,*]

[1]China-UK-NYNU-RRES Joint Laboratory of Insect Biology, Henan Key Laboratory of Insect Biology in Funiu Mountain, Nanyang Normal University (NYNU), Nanyang 473061, Henan, People's Republic of China

[2]Plant Division, Centre for Genetic Engineering and Biotechnology (CIGB), Havana 10600, Cuba

[3]Department of Life Sciences, Faculty of Natural Sciences, Imperial College London, Silwood Park campus, Ascot, Berkshire SL5 7PY, UK

[†]Equal contributors

*Correspondence and requests for materials: lidannytc@126.com (D.L.); cayrapardo73@163.com (CA-P)

Running title: *Proteomic and virulence of Btk haemolymph stimulon*

(a)

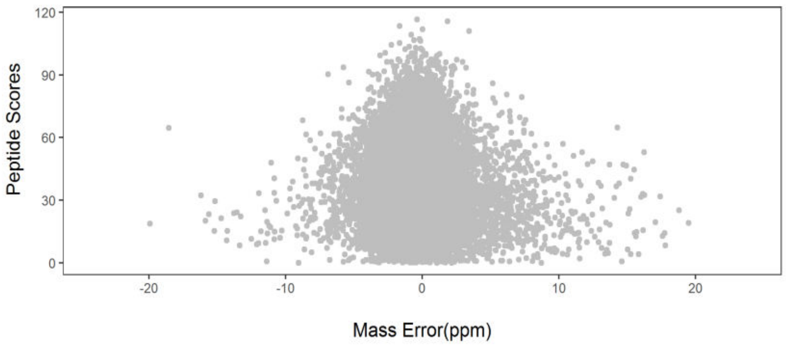

(b)

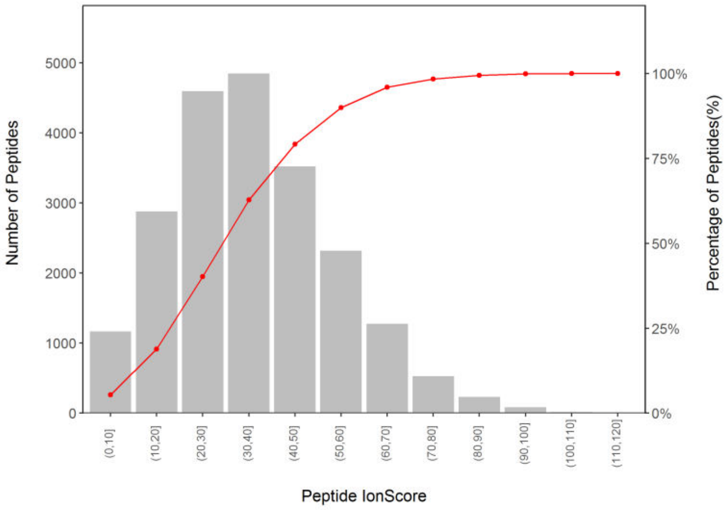

(c)

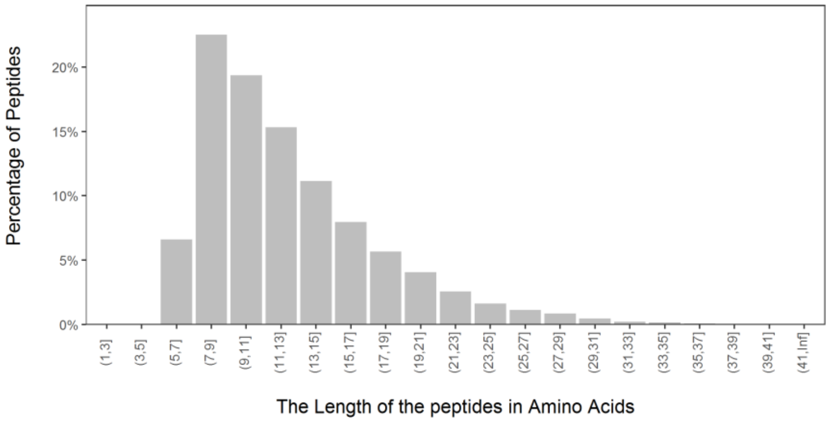

Figure S1. Quality control validation of MS data. (a) Mass error distribution. (b) Peptide ion score distribution. (c) Peptide length distribution.
